# Relationship between Conformation Traits and Lifetime Production Efficiency of Cows

**DOI:** 10.1155/2013/124690

**Published:** 2013-06-26

**Authors:** A. Sawa, M. Bogucki, S. Krężel-Czopek, W. Neja

**Affiliations:** Department of Cattle Breeding, University of Technology and Life Sciences, 85-084 Bydgoszcz, Poland

## Abstract

Analysis was made of the relationship between conformation traits and lifetime production efficiency of the cows that first calved in 2000 and represented the active population in the Pomorze and Kujawy regions of Poland. The CORR Pearson procedures of SAS package were used in the statistical calculations. It was found that there is a statistically significant relationship, weak or low on the Guilford scale, between conformation traits and lifetime production efficiency of the cows, which is slightly higher for milk yield than for longevity. The type and conformation traits appear to be more suitable than the detailed traits for predicting the lifetime production efficiency of cows. Lifetime performance was most strongly related to the overall score and udder score (*r* = 0.22), followed by the scores for type and conformation and legs and feet (*r* = 0.13), and detailed traits such as udder width and dairy character (*r* = 0.14). The highest positive effect on longevity was exerted by udder score and legs and feet (*r* = 0.11) and among detailed traits by udder placement (*r* = 0.14) and fore udder attachment (*r* = 0.10).

## 1. Introduction

The production efficiency of cows is closely related to milk yield in successive lactations and longevity. It is thought that longevity (which is measured in terms of lifespan, length of productive life, and survival to a certain age or calving) is one of the most important indicators of lifetime production efficiency of the cows. Cows with long productive lives have higher milk and milk component yields during their lives and give birth to more calves, and herd replacement costs decrease [1, 2], when summing up the results of cow longevity studies conducted in Polish research centres. Sawa [3] reported that cows in Poland are culled at 4.5 to 6.6 years of age, which is too early considering the natural lifespan of a cow (18–20 years) and that lifespan has a significant effect on the economic result of production [1]. A similar situation is found in Germany [4, 5]. Animal conformation is a very important trait, and in cattle it serves as a basis for selection of cows in breeding herds while accounting for the fact that cattle conformation is associated with production and nonproduction traits, and thus with production efficiency [6–11]. According to Guliński et al. [7], the conformation traits that are most related to dairy performance include udder width (*r* = 0.26), angularity (*r* = 0.21), overall score and type, and conformation (*r* = 0.19). A similar view is held by Chabuz et al. [12], who showed that milk yield was related most closely to conformation score on a 100-point scale (*r* = 0.43) and also to hip height (*r* = 0.31) and udder traits, in particular to udder width (*r* = 0.49) and fore udder extension (*r* = 0.35). Zavadilová and Štípková [11] showed that conformation traits are related to the length of cow's productive life and observed the closest relationships (*r* = −0.21 to −0.26) with capacity, body conformation, body depth, and rump width. The results of other studies conducted in the Czech Republic [13] show that most of the body traits had a slightly positive relationship to herd life, indicating that larger cows live longer. Cows with well-attached fore udder, high attached rear udder, strong central ligament, close front teat placement, and with moderately long teats showed the longest functional productive life (*P* < 0.05–0.001). Due to the transformation of local populations into a dairy type (in Poland, improvement of the Black-and-White Lowland breed with the Holstein-Friesian resulted in the Polish Black-and-White Holstein-Friesian breed, for which herd books have been kept since 2004), it is necessary to understand and improve the conformation traits (associated with the assessment of general and linear traits of type and conformation) that have the greatest impact on lifetime production efficiency of the cows. The conformation assessment system, which has been applied in Poland since 1996, follows the recommendations of international organizations ICAR and INTERBULL.

The aim of the study was to prove or reject the hypothesis that there is a relationship between conformation traits and lifetime production efficiency of the cows.

## 2. Material and Methods

The study used records from the SYMLEK system database concerning the conformation scores, milk yield in successive lactations, and longevity of 2214 Black-and-White cows improved with the Holstein-Friesian breed. The cows represented the active population in the Kujawsko-Pomorskie province (about 10% of the national population of performance recorded cows), which first calved in 2000. The following indicators of productive efficiency were accounted for milk yield in the first three complete lactations, lifetime milk yield (total yield from different complete lactations), lifespan (date of culling—date of birth), length of productive life (date of culling—date of first calving), and number of calvings throughout life. In 2000, the overall score for type and conformation of a dairy cow was calculated on a 100-point scale and included frame size (0–15 pts.), type and conformation (0–15 pts.), legs and feet (0–20 pts.), and udder (0–50 pts.). Detailed scoring accounted for the following traits, assessed on a scale of 1 to 9: body depth, chest width, rump angle, rump width, claws, rear leg set, fore udder attachment, rear udder attachment, medial suspensory ligament, udder placement, udder width, teat placement, teat length, dairy character, and hip height (cm). Statistical analysis included cows that were continuously evaluated. The CORR procedure of SAS [14] was used to estimate Pearson's simple phenotypic correlations between individual conformation traits and indicators of production efficiency of the cows. Due to the wide scoring scale used for linear traits (from 1 to 9) the data were not transformed, because the scores showed normal or near-normal distribution, which enabled statistical analyses to be performed without the need to normalize data distribution.

## 3. Results and Discussion

The relationships between conformation traits and lifetime production efficiency were analysed in the population of cows with an average milk yield in the first three lactations of 6333 kg, 6244 kg, and 6390 kg, lifetime milk yield of 20800 kg, lifespan of 5.80 years, and length of productive life of 3.44 years. The cows calved an average of 3.3 times during their lifetime.

Analysis of the results obtained (Table 1) shows that the scores for conformation traits of the investigated first-calf heifers are not substantially different from those reported for the Holstein-Friesian breed [15, 16]. For most linear traits, the analysed first-calf heifers received scores close to 5.5 pts. The highest average score was found for body depth (6.6 pts.) and the lowest for teat placement (4.5 pts.). Among the udder conformation traits, teat placement and length proved to be closest to the standard for the Holstein-Friesian breed. Variation in the scores for type and conformation was similar at several percent, while detailed traits showed high variation (20–29%).

The results obtained show the presence of weak or low relationships (mostly statistically significant) on the Guilford scale between individual conformation traits and indicators of lifetime production efficiency in the cows. Analysis of the relationships between individual conformation traits and indicators of lifetime production efficiency revealed that the correlations were slightly higher for the overall score for type and conformation traits than for detailed traits. In particular, these differences concerned the relationships between lifetime milk yield and score for type and conformation traits (*r* = 0.09–0.22) and detailed traits (*r* = 0.00–0.14). Therefore, the type and conformation traits appear to be more suitable for predicting the lifetime production efficiency of cows. Among the detailed traits, the most closely traits associated with lifetime milk yield were udder width (*r* = 0.14) and dairy character (*r* = 0.13).

Closer relationships were found more between conformation traits and milk performance indicators (*r* = 0.00 ± 0.40) than between conformation traits and longevity indicators (*r* = 0.00 ± 0.14). It is worth noting that the relationship between conformation traits and milk yield decreases as the milk production period progresses; in the first production cycle, the correlations ranged from *r* = 0.00 to *r* = ±0.40 and those for lifetime milk yield were between *r* = 0.00 and *r* = ±0.22. The findings of other authors [17, 18] also show that the correlations between conformation traits and milk and milk component yield tended to decrease in successive lactations.

The correlations between conformation traits and first lactation milk yield were at a similar level to those reported by Chabuz et al. [12]. Compared to ours, lower correlations between conformation traits and milk yield of first-calf heifers were obtained by Pawlina et al. [16] and higher by Kruszyński et al. [15]. Analysis of the relationship between conformation traits and milk yield of first-calf heifers showed that it was strongest (*r* = 0.20–0.40) for type and conformation traits such as overall score, type and conformation, udder, frame size, and for detailed traits such as udder width, dairy character, udder placement, hip height, body depth, and chest width. Kruszyński et al. [15] found the highest correlations between first lactation milk yield and frame size (*r* = 0.54), type and conformation (*r* = 0.51), hip height (*r* = 0.42), medial suspensory ligament (*r* = 0.43), and rear udder attachment (*r* = 0.29).

As already noted, the correlations between conformation traits and longevity indicators had low values (*r* = 0.00 ± 0.14). When analysing the relationships between conformation traits and longevity indicators, it was found that lifespan, length of productive life, and number of lactations depend primarily on the conformation of legs and feet (*r* = 0.11) and udder conformation (udder as a general trait in type and conformation score) (*r* = 0.11) and, among detailed traits, on udder placement (*r* = 0.14) and fore udder attachment (*r* = 0.10). The critical importance of leg conformation for the length of productive life in cows was also indicated by the findings of Cramer et al. [19]. The results obtained are in agreement with the findings of Strapák et al. [20], who observed that correlations of particular udder traits and functional length of productive life ranged from *r* = 0.1 (udder cleanness evaluated by nonlinear method) to *r* = 0.19 (suspensory ligament and teat conformation). In a study by Vacek et al. [13], the correlations between conformation traits and length of productive life ranged from −0.061 to 0.160, assuming the highest values for traits such as udder (*r* = 0.16), dairy character (*r* = 0.145), and feet and legs (0.126). Higher correlations than ours were estimated by Jairath et al. [21], who analysed a large number of data on first lactations and classification of type (896529 records) in the Holstein cows born from 1977 to 1991. A high genetic correlation was found between functional longevity and total score (*r* = 0.59), body frame (*r* = 0.20), rump angle (*r* = 0.19), feet and legs (*r* = 0.23), fore udder (*r* = 0.56), rear udder (*r* = 0.49), udder (*r* = 0.57), and dairy character (*r* = 0.06).

## 4. Conclusions

In summary, there is a statistically significant relationship, weak or low on the Guilford scale, between conformation traits and lifetime production efficiency of the cows, which is slightly higher for milk yield than for longevity. The type and conformation traits appear to be more suitable than the detailed traits for predicting the lifetime production efficiency of cows. Lifetime performance was most strongly related to the overall score and udder score (*r* = 0.22), followed by the scores for type and conformation and legs and feet (*r* = 0.13), and detailed traits such as udder width and dairy character (*r* = 0.14). The highest positive effect on longevity was exerted by udder score and legs and feet (*r* = 0.11), and among detailed traits by udder placement (*r* = 0.14) and fore udder attachment (*r* = 0.10). 

## Figures and Tables

**Table 1:** Mean scores for conformation traits and phenotypic correlations between conformation traits and production indicators of the cows.

Traits	Mean	Variation index	Yield milk (kg)				Length of ( years)		No. of calving
			I lactation	II lactation	III lactation	Performance life	life	productive	
General score for type and conformation	75,9	4,7	0,39^xx^	0,28^xx^	0,24^xx^	0,22^xx^	0,07^xx^	0,08^xx^	0,05^x^
Frame size	11,08	10,9	0,29^xx^	0,22^xx^	0,15^xx^	0,09^xx^	−0,03	−0,03	−0,05^x^
Type and conformation	10,84	7,7	0,35^xx^	0,23^xx^	0,19^xx^	0,14^xx^	0,01	0,01	−0,02
Legs and feet	15,04	5,5	0,01	0,06^xx^	0,05	0,12^xx^	0,09^xx^	0,11^xx^	0,11^xx^
Udder	39,01	5,8	0,32^xx^	0,21^xx^	0,21^xx^	0,21^xx^	0,10^xx^	0,11^xx^	0,08^xx^
Hip height	137,3	3,1	0,28^xx^	0,23^xx^	0,14^xx^	0,09^xx^	−0,03	−0,03	−0,05^x^

Features detailed									
Body depth	6,6	15,2	0,21^xx^	0,08^xx^	0,09^xx^	0,02	−0,04	−0,05^x^	−0,06^xx^
Chest width	5,1	24,5	0,20^xx^	0,17^xx^	0,07^xx^	0,03	−0,06^xx^	−0,07^xx^	−0,07^xx^
Rump angle	5,4	20,9	0,02	−0,00	−0,02	−0,00	−0,00	−0,00	0,00
Rump width	5,3	27,7	0,09^xx^	0,06^xx^	0,05	−0,01	−0,04	−0,06^x^	−0,06^xx^
Claws	4,8	28,3	0,10^xx^	0,09^xx^	0,09^xx^	0,07^xx^	0,01	0,01	0,01
Rear leg set—rear view	5,6	22,9	0,18^xx^	0,16^xx^	0,13^xx^	0,10^xx^	0,01	0,03	0,02
Fore udder attachment	5,4	25,7	−0,02	0,03	0,02	0,09^xx^	0,11^xx^	0,10^xx^	0,10^xx^
Rear udder attachment	5,0	24,4	0,15^xx^	0,08^xx^	0,10^xx^	0,09^xx^	0,04	0,05^x^	0,02
Medial suspensory ligament	5,2	27,7	0,06^xx^	0,01	0,06^x^	0,09^xx^	0,05	0,08^xx^	0,09^xx^
Udder placement	5,7	28,2	−0,20^xx^	−0,09^xx^	−0,05	0,06^x^	0,14^xx^	0,14^xx^	0,14^xx^
Udder width	5,4	21,6	0,40^xx^	0,26^xx^	0,19^xx^	0,14^xx^	−0,01	−0,01	−0,03
Teat placement	4,5	29,4	0,06^xx^	0,03	0,09^xx^	0,02	−0,00	0,00	−0,01
Teat length	4,8	26,9	−0,06^xx^	−0,09^xx^	−0,11^xx^	−0,07^xx^	−0,02	−0,03	−0,02
Dairy character	5,5	22,2	0,30^xx^	0,15^xx^	0,17^xx^	0,13^xx^	0,03	0,04	0,01

^xx^Significance at *P *≤ 0.01, ^x^significance at *P *≤ 0.05.
